# Innovative approach to designing user-centred digital solutions for plastic surgery patients with non-melanoma skin cancer

**DOI:** 10.3389/fpubh.2025.1685882

**Published:** 2025-11-17

**Authors:** Annachiara Cavaliere, Vincenzo De Luca, Michele Virgolesi, Guido Iaccarino, Lorenzo Mercurio, Maddalena Illario, Fabrizio Schonauer

**Affiliations:** 1 Dipartimento di Sanità Pubblica, Università degli Studi di Napoli Federico II, Napoli, Italy; 2 Dipartimento di Medicina Clinica e Chirurgia, Università degli Studi di Napoli Federico II, Napoli, Italy

**Keywords:** non-melanoma skin cancers, plastic surgery, mHealth, digital health, user-centred design, telemedicine, telehealth, Persona

## Abstract

Non-melanoma skin cancers (NMSCs) are the most common malignancy in fair-skinned patients. NMSCs incidence increases with age, and as the worldwide population is constantly ageing, the management of patients with NMSCs poses a considerable burden on healthcare systems. This study aimed to identify key digital solutions addressing the unmet needs of individuals with a NMSCs profile, through an adaptation of the “Blueprint on Digital Transformation in Health and Care in an Ageing Society” (Blueprint) user-centred design methodology. A phone survey was administered to 87 patients treated at the Plastic Surgery Unit of Federico II University Hospital from 2022 to 2023, to analyse the demographic, clinical and social characteristics of NMSCs patients. The theoretical elaboration of NMSCs personas was developed by an interdisciplinary Focus Group (FG). Seventy patients agreed to participate in the survey when contacted by phone, 55% were males. Only 4 patients had primary education. 52 patients (74%) had BCC, and 18 patients (26%) presented SCC. Most frequently referred comorbidities were diabetes (17%), hypertension (28%), overweight (41%) and obesity (13%). Patients’ primary concern was the possible need for extended hospitalization and not having easily accessible information and medical assistance. 92% of our patients declared that they could rely on someone to assist them in using a smartphone or a tablet. The FG elaborated a “Persona” named Pino, a 67-year-old retired man living in the suburban Neapolitan area. He suffers from diabetes and hypertension. Pino is also overweight, smokes a few cigarettes a day and occasionally drinks wine or spirits. He has a diagnosis of an invasive basal cell carcinoma of the scalp. Pino has trouble keeping track of all the preoperative and postoperative prescriptions and would like to have direct contact with the healthcare practitioners. He has difficulties adhering to his therapy. Pino is unable to reproduce postoperative dressings correctly. The digital solution proposed by the FG is a mHealth solution that supports the patient with a shared digital calendar for medical appointments, connected to a smart pillbox. The solution must support patients through a digital education package that includes tutorials on dressing and dressing prescriptions. An AI-supported chatbot would allow patients to quickly access the information they need. The Blueprint “Persona” methodology is a powerful tool to help healthcare professionals identify the unmet needs of specific patient subsets. Further studies are necessary to evaluate the actual feasibility of implementing therapeutic pathways for patients with NMSCs through mHealth solutions.

## 1 Introduction

Skin cancer, including melanoma, basal cell carcinoma and cutaneous squamous cell carcinoma, has one of the highest global incidences (1). “Non-melanoma skin cancers” (NMSCs) include squamous cell carcinoma (SCC) and basal cell carcinoma (BCC). NMSCs represent approximately 1/3 of all cancers diagnosed worldwide yearly and are the most common malignancies in fair-skinned patients (2, 3, 38). BCCs account for approximately 80% of all NMSCs, while SCC represents approximately 20% (3). The World Health Organisation (WHO) estimated 2–3 million cases per year (4). However, this figure may be underestimated, as a more recent study reported approximately 3.5 million cases per year in the United States (5). NMSCs are associated with extremely low mortality rates and are infrequently metastatic. Nevertheless, they can lead to significant morbidity for patients, causing local destruction and sometimes disfigurement (6, 7). The incidence of NMSCs is reported to increase with age, and mortality is rare in the general population. As the worldwide population is constantly ageing, the management of patients with NMSCs poses a considerable burden on the healthcare system (8–10). Perioperative management of patients with NMSCs can be challenging and requires continuous interaction between patients and healthcare professionals. During the preoperative phase, failing to follow the prescriptions, such as therapy adjustments or further diagnostic examination, may delay the surgery, with serious consequences for patients. Timing in NMSC treatment is crucial, as these cancers may grow rapidly and become deeply invasive, thereby requiring more complex surgeries, with the risk of severe disfigurement or, in the worst case, becoming inoperable (7). After surgery, patients must follow all instructions and prescriptions provided by the surgeon. Not adhering to this instruction may lead to unfavourable outcomes and postoperative complications, prolonging healing times and increasing the risk of the wound becoming chronic, which can negatively affect patients’ autonomy, social life and quality of life (11). Moreover, surgery delays, complications and unfavourable outcomes contribute to the increasing economic burden associated with NMSC treatment (9, 10). Patients with NMSCs, especially those with multiple comorbidities, may benefit from specifically designed mobile health (mHealth) solutions, such as an mHealth app that is user-friendly, integrated and easily accessible. This helps them adhere to preoperative and postoperative indications and allows them to stay in continuous contact with the healthcare professionals involved. Digital health services can enhance health outcomes by improving medical diagnosis, facilitating data-driven treatment decisions, promoting digital therapy and enhancing clinical trials (12). Digital solutions improve disease self-management and person-centred care (13), and they create more evidence-based knowledge, skills and competencies for healthcare professionals to support healthcare based on technologies such as the Internet of Things (IoT), virtual assistance, remote monitoring, artificial intelligence, big data analytics, smart wearables and platforms, which allow continuous and integrated care (14). Mobile health technology (mHealth) is an emerging reality helping in the delivery of healthcare through mobile communication technologies (such as mobile apps) (15, 16). mHealth, including chatbots powered by artificial intelligence, has proven to be a good alternative to provide high-quality healthcare services to different patient populations, especially patients with low incomes and who live in remote places (far from reference centres), thereby making healthcare more accessible and affordable for all (17). The large-scale implementation of digital solutions is hindered by organisational, technical, economic and cultural barriers (18), which require tailored adaptations to be integrated into current care services (19, 20). The European Blueprint on Digital Transformation of Health and Care for the Ageing Society, part of the European Innovation Partnership on Active and Healthy Ageing (EIP AHA), is a European Commission initiative designed to promote investments in digital innovation for active and healthy ageing across a large number of European regions. The Blueprint aims to ensure the engagement of stakeholders, particularly SMEs and public entities, in the co-development of innovative solutions addressing the unmet needs of patients and older adults, thereby advancing the digital transformation of health and care (21).

The present study aimed to identify, through a user-centred design method, the social and health-related unmet needs of patients with NMSCs to remodel the diagnostic-therapeutic pathways by introducing digital technologies into healthcare service provision. The user case scenario derived from this preliminary study will be essential for the further development of a specifically designed mHealth app for the perioperative management of patients with NMSCs.

## 2 Materials and methods

The study adapted the European Commission’s “Blueprint on Digital Transformation of Health and Care for the Ageing Society” (Blueprint) methodology through a mixed qualitative–quantitative approach aimed at identifying and specifying key digital solutions and high-impact user scenarios for patients diagnosed with BCC and SCC who were referred to the Plastic Surgery Department of Federico II University Hospital in Naples, Italy (22). The Blueprint “Personas” methodology is a patient-centred approach specifically designed to highlight patients’ unmet needs to design key digital solutions and usage scenarios (22, 23). The “Persona” is a single, specific, hypothetical patient who can represent a specific patient population (24). The outlined “Persona” has a realistic name, an avatar, and a brief description of his/her needs, goals, hopes, dreams and attitudes. Behavioural characteristics are included as they may affect the results of the proposed intervention in the short and long term.

The involvement of the most experienced professionals in healthcare services provided to patients with NMSCs was ensured through the establishment of a Focus Group (FG), which aimed to identify care processes requiring change through the use of IT (25, 26). The interdisciplinary FG included the following professionals:

- n. 2 plastic surgeons.
- n. 1 dermatologist.
- n. 1 nurse.
- n. 2 experts in digital health.

The FG discussion was divided into four in-person meetings held between September 2022 and July 2023 to address the following topics: (i) methodology design and approval; (ii) analysis of the phone survey results and design of the Persona use case; (iii) identification of applicable digital solutions and datasets; and (iv) content approval. A rapporteur documented the content of the meetings and extracted the relevant information, recording it in an electronic form. The FG members approved each item recorded on the electronic form. In case of discrepancies, the transcripts were analysed thematically. In addition, two researchers independently coded the material, and discrepancies were resolved by consensus with a third senior researcher in the specific discipline.

### 2.1 Phone survey

**Figure 1:**
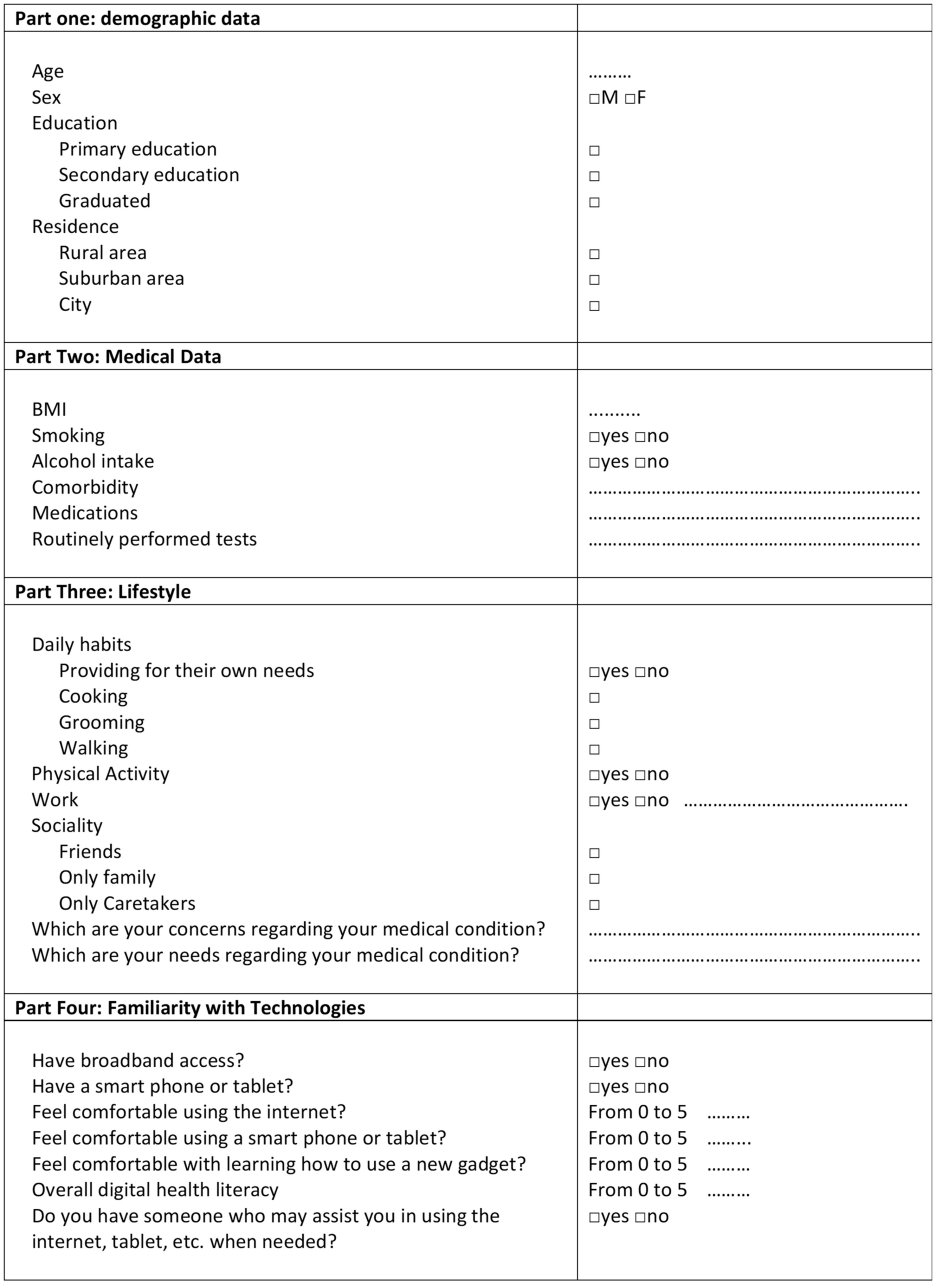
Phone survey used to collect patients’ demographic data, medical conditions, daily habits and familiarity with new technologies.

A phone survey (Figure 1) was administered anonymously to a group of patients to self-assess their health needs and the support available for managing their health conditions. The survey was divided into four sections. The first section assessed patients’ demographic data, including age, gender, education and residence (rural, suburban, or urban area). The second section assessed medical data: BMI, smoking habits, alcohol intake (considered at risk if >2 units/day for men, >1 units/day for women, or >1 units/day for people aged >65 years) (27), comorbidity, medications and routinely performed medical tests. The third section assessed daily habits, social environment, needs and concerns. The last section assessed patients’ familiarity with new technologies (22). To develop the questionnaire, closed-ended questions and a 5-point Likert scale were used as a psychometric tool to evaluate users’ opinions regarding their confidence in using IT and their general level of IT literacy. The data were processed in accordance with Art. 89 of the General Data Protection Regulation, which allows the processing of personal data for archiving purposes in the public interest, scientific or historical research or statistical purposes, provided that technical and organisational measures are implemented to ensure the principle of data minimisation (39). During the follow-up visit, before contacting patients by phone, informed consent was obtained from all participants in accordance with clinical practice. Statistical analysis was conducted on anonymised data, and no data were used, nor were references made to a specific single patient. Only researchers from the Plastic Surgery Unit of Federico II University Hospital in Naples who were involved in this study stored the personal data used for research purposes, using identification codes. The inclusion criteria included patients aged 50–90 years, of any gender, residing in the Campania region (Italy), referred to the Plastic Surgery Unit of Federico II University Hospital and diagnosed with NMSCs. Since this was not a clinical study and the data were anonymised and processed in accordance with current data protection regulations, the protocol was not submitted to the ethics committee for approval.

### 2.2 Blueprint Persona elicitation

Based on the results of the survey, a theoretical elaboration of the Blueprint Persona was developed by the FG. The healthcare professionals involved in the FG (plastic surgeons, dermatologists, and nurses) iteratively answered the questions in the Blueprint Persona Development tool (Supplementary material) and agreed on the final response. The Blueprint Persona Development tool allowed us to break down the Persona characteristics into five components. The “Introduction” section included the Persona’s name, age, current life stage and a classification of their needs based on complexity, level of digital literacy (e.g., Internet and mobile technology use) and living context (urban, suburban, or rural area). The “Digital Skills” section listed the Persona’s skills, ranging from minimal to advanced, in areas such as online navigation, mobile technology proficiency, enthusiasm for new technologies, understanding digital health information and the need for support with information and communication technologies. The “Personal History” section provided a brief account of the Persona’s life, highlighting personal facts, emotions, daily responsibilities, essential needs and challenges they have encountered. The “Overview” section was divided into eight subsections: “What is significant to the patient,” “Daily activities,” “Own resources and support systems,” “Key events, concerns and personal problems,” and “Health,” among other areas that explored the distinctive aspects of the patient’s life. The “Needs” section summarised the unmet needs that can be addressed through IT-supported intervention.

### 2.3 Design exercise of a digital solution

The FG further discussed and identified significant unmet needs to be addressed, reporting the current services provided at Federico II University Hospital. The experts in digital health translated the current services into applicable digital health solutions.

## 3 Results

### 3.1 Phone survey results

The phone survey was administered to 87 patients who were referred to the Plastic Surgery Unit of Federico II University Hospital and had signed the informed consent to participate in the study. A total of 70 patients agreed to participate in the survey when contacted by phone. The group consisted of 39 men and 31 women, with a mean age of 67 ± 8.0 years (range 53–87 years). The patients’ education level was reported as follows: 29 patients were graduates (42%), 37 patients had secondary education (51%) and four patients had primary education (7%). In total, 31 patients (45%) lived in the city, 37 patients (52%) lived in suburban areas and two patients (3%) lived in rural areas. In our group, 52 patients (74%) had BCC and 18 patients (26%) presented SCC. Most frequently referred comorbidities were diabetes (17%), hypertension (28%), overweight (41%) and obesity (13%). A total of 17% of the patients were active smokers, and only 1% reported high alcohol consumption. In our group, 27 patients were active workers (17 M, 10 F), 34 patients were retired (22 M, 12 F) and nine were housewives. Concerning the social environment, 41% of the patients reported having an active social life, 48% reported having only family as their social contacts and 11% reported having no social life at all (excluding caregivers). For most of our patients, their primary concern about their condition was the potential need for extended hospitalisation and recovery. The most frequently reported need was access to easily available information and medical assistance. The last part of the questionnaire assessed the patients’ familiarity with new technologies. In our group, 84% had broadband access at home and 92% of them owned a smartphone or a tablet. The mean reported values on a 5-point Likert scale assessing the patients’ comfort with new technologies were 3.7 for “Feel comfortable using the internet,” 3.2 for “Feel comfortable using a smart phone or tablet” and 2.3 for “Feel comfortable learning how to use a new gadget.” The reported “Overall digital health literacy” mean value was 1.1. Furthermore, 92% of our patients reported that they could rely on someone (sons, caregivers, or neighbours) to assist them in using a smartphone or a tablet. The overall variability of the observed percentages was characterised by a variance of 643.0 and a standard deviation (SD) of 25.4. The greatest variability was observed for carcinoma type (SD = 33.9), followed by area of residence (SD = 26.5) and education level (SD = 23.2). Intermediate variability was observed for social contacts (SD = 19.7) and occupational status (SD = 17.5), whereas the lowest values were found for habits (SD = 11.3), comorbidities (SD = 12.6) and technology use (SD = 5.7) (Table 1).

**Table 1:** Phone survey results.

**Characteristic**	**Value**
Demographic data	
Age (years), mean (SD)	67 (8.0)
Gender, male, n (%)	39 (55.7)
Minimum Age in years	53
Maximum Age in years	87
Level of education, S(SD)	540.3 (23.2)
Primary School or No Education, n(%)	4 (5.7)
Secondary School, n(%)	37 (52.8)
University Degree, n(%)	29 (41.4)
Residence, S(SD)	702.3 (26.5)
Rural Area, n(%)	2 (2.8)
Suburban Area, n(%)	37 (52.8)
City, n(%)	31 (44.2)
Occupation, S(SD)	305.3 (17.5)
Active workers, n(%)	27(38.5)
Retired, n(%)	34(48.5)
Housewives, n(%)	9 (12.8)
Sociality, S(SD)	386.3 (19.7)
Active social life	29 (41.4)
Only family	33 (47.1)
No social life	8 (11.4)
Medical data	
Type of skin, S(SD)	1152.0 (33.9)
BCC, n(%)	52 (74.2)
SCC, n(%)	18 (25.7)
Comorbidities, S(SD)	157.6 (12.6)
Overweight, n(%)	29 (41.4)
Hypertension, n(%)	20 (28.5)
Diabetes, n(%)	12 (17.1)
Obesity, n(%)	9 (12.8)
Lifestyle, S(SD)	128.0 (11.3)
Smoking	12 (17.1)
High Alcohol intake	1 (1.4)
Digital Literacy	
Familiarity with Technologies, S(SD)	32.0 (5.6)
Broadband access	59 (84.2)
Smart phone or tablet	65 (92.8)
Comfort with digital technologies (score 1-5)	
Internet use, mean(SD)	3.7 (1.2)
Smartphone or tables use, mean(SD)	3.2 (1.4)
Learning how to use a new gadget, mean(SD)	2.3 (1.1)
Overall digital health literacy, mean(SD)	1.1 (0.9)
Having someone who may assist them using technologies, n(%)	65 (92.8)

### 3.2 NMSC Persona profile

**Figure 2:**
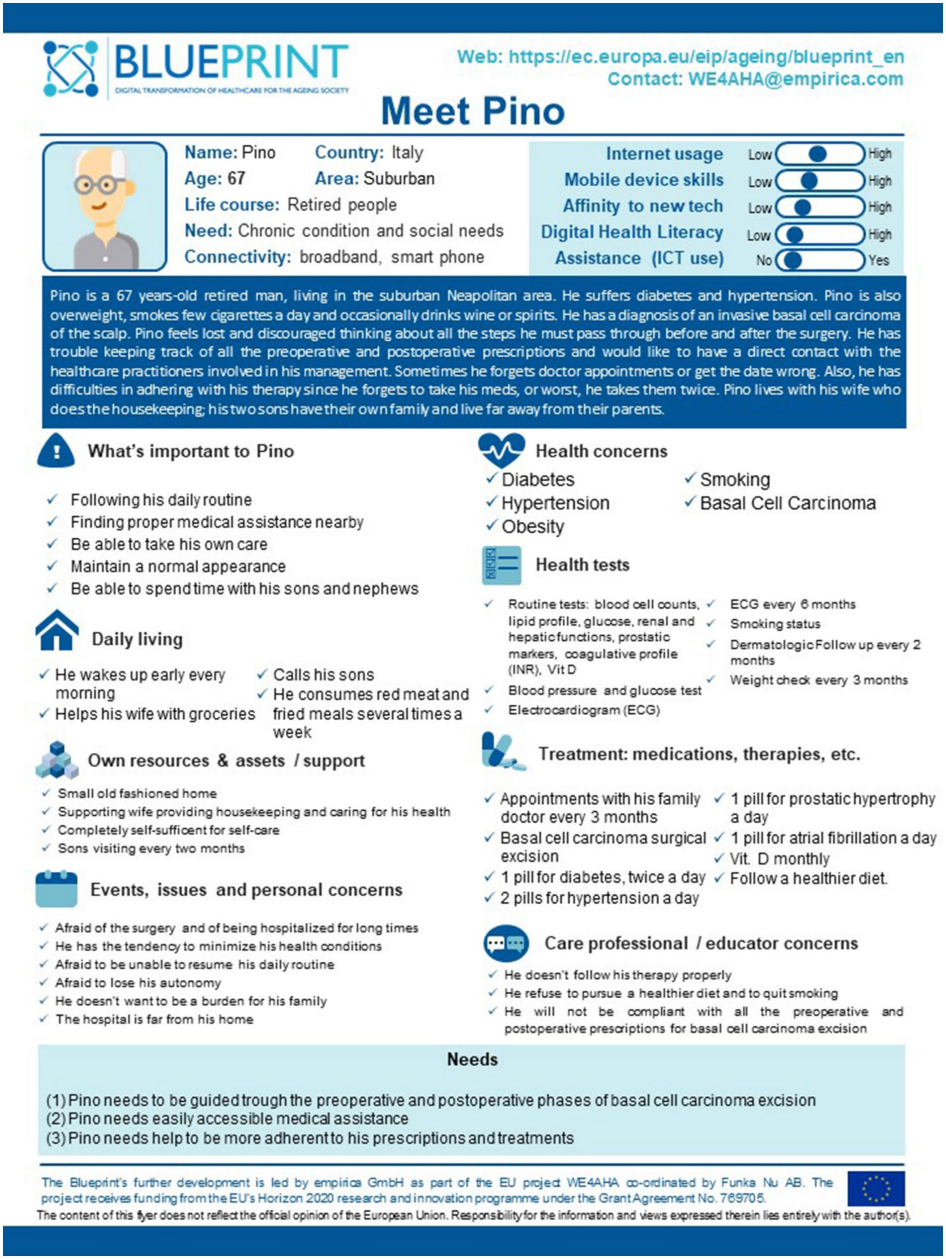
Pino’s Blueprint Persona profile card.

Finally, a Blueprint “Persona” named Pino, embodying all the characteristics, recurring issues and needs reported in the survey, was developed (Figure 2). Pino is a 67-year-old retired man living in the suburban Neapolitan area. He suffers from diabetes and hypertension. Pino is also overweight, smokes a few cigarettes a day and occasionally drinks wine or spirits. He has a diagnosis of an invasive basal cell carcinoma of the scalp. Pino feels lost and discouraged when thinking about all the steps he must go through before and after surgery. He has trouble keeping track of all the preoperative and postoperative prescriptions and would like to have direct contact with the healthcare practitioners involved in his management. Sometimes he forgets doctor appointments or gets the date wrong. In addition, he has difficulties adhering to his therapy since he may forget to take medications or, worse, take them twice. Although postoperative dressings are relatively simple, Pino is unable to perform them correctly, and his healing process is taking longer than usual. What emerged from the FG discussion was that complex patients such as Pino will benefit from specifically designed mHealth solutions to address their unmet needs.

### 3.3 Applicable digital health solution

The digital solution proposed by the digital health experts of the FG could be implemented in a user-friendly, integrated and easily accessible mHealth app, helping patients address their unmet needs (Table 2). An mHealth solution that supports the patient must include a shared digital calendar with alerts for consultations and follow-up appointments and a shared treatment plan that can be updated remotely. The solution should also include alerts for medication times and be connected via Bluetooth to a smart pillbox, which allows the patient to take the correct medication at the right time and in the correct dose. The mHealth solution must support patients through a digital education package that includes a video library with tutorials on dressings and dressing prescriptions, guidelines, photos of the necessary products and instructions on how to use them. It is crucial that the mHealth solution provides a list of FAQs. It needs to be accessible to patients and linked to video tutorials and informational materials. An AI-supported chatbot would allow patients to quickly access the information they need. Table 3 outlines the links between current clinical tasks, unmet needs and the potential digital tools that can support patients and professionals. Table 4 presents the structured digital intervention dataset, mapping each identified unmet need to relevant clinical and social data, digital tools, care settings and interoperability requirements. This mapping supports the future co-design and implementation of tailored mHealth pathways for patients with NMSCs.

**Table 2:** Key features of applicable digital health solution.

Identified unmet needs	Current services	Applicable digital solutions
Attending preoperative consultations	Healthcare professionals define a consultation calendar which is communicated to the patient orally and printed on a paper	Shared digital calendar, with alerts via SMS or mHealth app with personalized information (time, date, what to bring...) and indications on patient preparation (e.g., fasting hours)
Polypharmacy management	Health professionals draw up a personalized treatment plan on taking medications which is communicated orally to the patient and printed on a paper	Shared therapeutic plan, which can be updated remotely, with alerts on drug intake times and connected via Bluetooth to a smart pill box, which allows the patient to take the right drug, at the correct time and in the correct doses.
Adherence to postoperative dressing prescriptions	Healthcare professionals give the patient a sheet with post-operative recommendations and a checklist of actions to be carried out. Healthcare Professionals communicate orally dressing prescriptions.	Digital training package including a videolibrary with dressings tutorials and dressing prescriptions with guidelines and photos of the products they need and how to use them
Follow up and recurrence prevention	Health professionals provide a paper follow-up appointment reminder and tips for early detection of recurrences orally and through paper guidelines	Shared digital calendar, with alerts for follow-up appointments and a video tutorial for preventing recurrences, with key information and photo examples. A notification system promotes patient engagement in preventing relapses
Easily accessible medical assistance	Patients communicate their doubts and information requests by calling the hospital or writing directly to professionals via SMS.	A list of F.A.Q. is accessible by the patient with video tutorials and information materials. An AI-supported chatbot allows patients to quickly access the information they require.

**Table 3:** Summary table of identified technological solutions.

Use case domains	Sub-category	Identified unmet needs	Current clinical tasks	Functionalities of the supportive digital solutions	Examples
Professionals/carers concerns	Communication and monitoring	Lack of continuous direct contact between patient and care team	Postoperative follow-up, treatment education	AI chatbot, educational videos, shared digital calendar	Integrated mHealth app with reminders and FAQs
	Telemedicine	Need for remote consultations, especially in suburban areas	Post-op monitoring, easier access to professionals	Video calls, asynchronous messaging system	Secure telemedicine platform, virtual follow-up
Health concerns	Hospitalization and complications	Fear of prolonged hospital stay, infections, slow healing	Wound healing monitoring, complication management	Remote monitoring, digital educational packages	Photo documentation via app for wound tracking
	Recurrence	Concern about future lesions or recurrence	Periodic dermatological follow-ups	Follow-up reminders, educational content on recurrence signs	App-based guided self-examination
Treatments	Therapy adherence	Forgetting medications or incorrect dosage	Medication management, follow-ups	Medication alerts, Bluetooth-connected pillbox, shared treatment plan	Bluetooth-connected pillbox with smart alerts
	Postoperative care	Difficulty following dressing instructions and recommendations	Health education, self-care support	Video tutorials, personalized care instructions, FAQs	Illustrated material, app notifications
Daily living	Daily organization	Difficulty remembering appointments, coordinating tasks	Reminders, daily planning	Shared calendar with alerts	App synchronized with caregiver’s phone
	Autonomy support	Loss of independence after surgery	Discharge planning and home recovery	Personalized reminders and recovery checklist	Recovery checklists integrated in app
What is important to patient	Access to information	Need for clear, updated, and accessible medical information	Patient education and empowerment	Video library, interactive materials, FAQs	App with chatbot and visual self-guidance
	Safety and continuity of care	Feeling abandoned after discharge	Maintaining contact with healthcare team	Asynchronous communication, remote monitoring	Messaging system with nurse/surgeon
Digital engagement and support	Digital support and navigation	Low confidence with tech use, reliance on caregiver support	App usage, tech onboarding	Simple UI, caregiver-assisted navigation	Basic digital health literacy; tech support from family and caregivers
Own resources and assets	Geographic and social support	Support exists but not integrated	Caregiver involvement in care process	Shared app access, dual notifications	Suburban area; caregivers involved in tech use

**Table 4:** Digital intervention data set.

Unmet needs	Data	Tools	Settings	Interoperability	Notes
Improving accessibility to healthcare	Area of residence (urban, suburban, rural); device ownership (smartphone/tablet)	mHealth app, chatbot, video tutorials, telemedicine portal	Outpatient and home settings	Compatible with electronic health records	92% own a smartphone/tablet; 84% broadband; 52% in suburban areas
Improving ability to self-manage disease	Therapy adherence; understanding medical instructions	Shared calendar, Bluetooth smart pillbox, digital education	Preoperative and postoperative settings	Sync with Bluetooth devices, data storage	Basic digital health literacy; tech support from family and caregivers
Polypharmacy management and adherence	Medication intake routines, risk of mismanagement	Medication reminders, smart pillbox	Home setting	Pharmacy or EHR system integration	Frequent medication intake errors reported
Remote consultations (telemedicine)	Need for medical follow-up without in-person visits	Video calls, secure messaging systems	Home and outpatient	Integration with EHRs and hospital platforms	Relevant due to geographic dispersion (suburban areas)
Adherence to a healthy lifestyle	Smoking, alcohol use, overweight	Educational videos, motivational content	Home	Optional connection to lifestyle tracking tools	Pino smokes, is overweight, drinks occasionally
Digital engagement and support	Low tech confidence, high reliance on others	User-friendly app, caregiver-linked access	Home	User-centred, adaptive design	Basic digital health literacy; tech support from family and caregivers

## 4 Discussion

The number of patients diagnosed with NMSCs is constantly increasing, posing a considerable burden on the healthcare system (8–10). These patients are typically older adults (>65 years) who are affected by several comorbidities and associated with increased frailty in different domains (7, 28, 29). The Blueprint “Personas” approach, developed by the “Blueprint on Digital Transformation in Health and Care in an Ageing Society,” is a synthetic and intuitive inference process that makes it easier to identify the unmet needs of a specific subset (24). As indicated by our results, patients diagnosed with NMSCs require a multidisciplinary approach that focuses not only on one-time disease treatment but also on recurrence prevention and lifestyle improvement. MHealth may influence patients’ attitudes and behaviour, facilitating information exchange between patients and healthcare professionals (30, 31). MHealth apps have proven effective in providing high-quality healthcare assistance in the management of chronic diseases (e.g., diabetes, asthma and hypertension), and various apps have been developed so far (14, 24). Nevertheless, still few mHealth solutions have been developed for surgical settings, either preoperative or postoperative settings (32–34). Through the Blueprint “Persona” methodology, we aim to develop an adapted mHealth app to help patients with NMSCs navigate through the diagnostic and therapeutic pathway and, in the long term, to improve their lifestyle. This customizable app will provide personalised information and recommendations to patients, including the date and time of surgery, the need for their usual therapy adjustments (e.g., oral anticoagulant suspension) and eventual multidisciplinary preoperative or postoperative consultations. This can help patients better understand the surgical procedure, its risks and potential outcomes. Recent studies have shown that patients who received preoperative training using a digital platform reported lower levels of anxiety and pain and higher levels of satisfaction than those who received traditional training (35). Thanks to the interactive nature of the apps in general, this app may also monitor patients’ compliance with preoperative and postoperative recommendations, reminding patients of their medication schedule, offering exercises to prevent complications and allowing patients to report any symptoms or concerns. Furthermore, patients will have the possibility to easily communicate with the different healthcare professionals involved in their multidisciplinary management through the app, thereby facilitating intervention in the case of complications and adverse events. Since patients with NMSCs may experience recurrence, the app will also be used for long-term follow-up, helping patients recognise the signs of potential recurrence and assisting them in scheduling their periodic dermatological check-ups. Patients who used a mobile app to report their symptoms after surgery showed a significantly lower rate of hospitalisation and emergency room visits than those who received standard care (36). The limited sample size in the studies reviewed and the scarcity of information on barriers to implementation suggest that further randomised controlled trials are needed to validate the solutions with respect to clinical and organisational outcomes. Nevertheless, we acknowledge that the adoption of digital services and solutions is invariably influenced by the socio-economic context, skills and integration gaps, both technological and organisational (37). Although our approach is designed to address the unmet needs of a real subset of patients, it will certainly require further validation through an implementation protocol.

## 5 Conclusion

The Blueprint “Persona” methodology is a powerful tool to help healthcare professionals identify the unmet needs of specific patient subsets. Through this approach, we developed an mHealth solution to help patients with NMSCs in addressing their needs, either in the preoperative or postoperative setting. Nevertheless, further studies are needed to evaluate the actual feasibility of implementing therapeutic pathways for patients with NMSCs using mHealth solutions.

## Data Availability

The raw data supporting the conclusions of this article will be made available by the authors, without undue reservation.
