# Supplementary material for: Innovative approach to designing user-centred digital solutions for plastic surgery patients with non-melanoma skin cancer
Source: Front Public Health. 2025 Nov 17;13:1685882. doi: 10.3389/fpubh.2025.1685882 (PMC12665651; doi:10.3389/fpubh.2025.1685882)
Supplement: Supplementary file 1 [file Table_1.DOCX]

Template for personas in the health and care needs over the life course framework and matrix*

**Matrix found at the end of the document*

| Name: | Age: |
| --- | --- |
| Life course   - Babies/children/young people - Working age adults - Retired people - Very old people 80+   Need:   - Generally well / good wellbeing - Chronic conditions and social needs - Chronic conditions - Social Needs - Complex needs | |
| Profile Summary  *(Age, situation, personal life, hobbies, health concerns, etc)* | |
| What’s Important to … | Own Resources & Assets / Support (not ICT-based)  *What forms of support (not ICT-based) does your persona already have access to? (Examples are: housing; support from family, friends, professionals (medical, legal, anyone who can help); personal assets/attributes e.g. being friendly and polite, sense of humour; social life incl. membership in organizations, etc) ability or willingness to self-care* |
| Daily living | Health concerns  *Focus also on RISKS and Justify your suspect based on molecular pathway* |
| Events, issues and personal concerns | Treatment: medications, therapies, etc |
| Health tests | Care professional concerns |
| Social care | Employment concerns |
| Technology-related resources  *(incl. access to and availability of such resources, capability to use them, etc)*  **Please answer the following guiding questions (7 in total):**  Does the persona…   1. Have broadband access? 2. Have a smart phone or tablet? 3. Feel comfortable using the internet? Yes   **On a scale of 0 – 5,** please rate (approx.) degree of comfort **(5 means very comfortable, no need for external support)**  **Rating:**   1. Feel comfortable using a smart phone or tablet?   **On a scale of 0 – 5,** please rate (approx.) degree of comfort. **Rating:**   1. Feel comfortable with learning how to use a new gadget?   **On a scale of 0 – 5,** please rate (approx.) degree of comfort.  **Rating:**   1. **Overall digital health literacy* - Please rate (approx.) [scale of 0 -5; 5 as high literacy]. Rating:**   **Digital health literacy definition: the ability to seek, find, understand and appraise health information from electronic resources and apply the knowledge gained to addressing or solving a health problem. (Source: Norman and Skinner, 2006 DOI:10.2196/jmir.8.2.e)*   1. Does persona have someone who will assist them in using the internet, tablet, etc when needed? No, but she’s rather autonomous   **Optional: Please insert any additional comments in the pink box below**: | Educational interventions / concerns |
| Technology-based solutions incl. ICT  *Please list any other forms of ICT-based support not mentioned above that would provide better health and care for the persona / target the needs of the persona.* | |
| Identified unmet needs | |

Reminder:

The boxes below may be used to describe particular aspects of your persona in more detail.

**Examples of possible additional fields/boxes:** socio-economic context, transportation, housing services, other concerns that are not health-related, etc

| Other (please specify)   - ... - ... - ... | Other (please specify)   - ... - ... - ... |
| --- | --- |
| Other (please specify)   - ... - ... - ... | Other (please specify)   - ... - ... - ... |
| Other (please specify)   - ... - ... - ... | Other (please specify)   - ... - ... - ... |
