# Supplementary material for: Innovative approach to designing user-centred digital solutions for plastic surgery patients with non-melanoma skin cancer
Source: Front Public Health. 2025 Nov 17;13:1685882. doi: 10.3389/fpubh.2025.1685882 (PMC12665651; doi:10.3389/fpubh.2025.1685882)
Supplement: Supplementary file 2 [file Table_2.DOCX]

# Table 1 – Phone survey results

| **Demographic data** |  |
| --- | --- |
| Age    Sex    Education  *Primary education*  *Secondary education*  *Graduated*    Residence  *Rural area*  *Suburban area*  *City* | 53-87 (mean 67±8.0)    39M 31F     (s^2^: 540,3 / s: 23,2)  4 *(7%)*  37 *(51%)*  29 *(42%)*    (s^2^: 702,3 / s: 26,5)  2 *(3%)*  37 *(52%)*  31 *(45%)* |
| **Medical Data** |  |
| BCC  SCC    Comorbidities  *Overweight*  *Hypertension*  *Diabetes*  *Obesity*    Smoking  High alcohol intake | (s^2^: 1152,0 / s: 33,9)  52 *(74%)*  18 *(26%)*    (s^2^: 157,6 / s: 12,6)  41%  28%  17%  13%   (s^2^: 128,0 / s: 11,3)  17%  1% |
| **Lifestyle** |  |
| Occupation  Active workers  Retired  Housewives    Sociality  *Active social life*  *Only family*  *No social life* | (s^2^: 305,3 / s: 17,5)  27 *(38%)*  34 *(48%)*  9 *(14%)*    (s^2^: 386,3 / s: 19,7)  *41%*  48%  11% |
| **Familiarity with Technologies** |  |
| Broadband access  *Smart phone or tablet*    Comfort with new technologies  *Internet use*  *Smart phone or tablet use*  *learning how to use a new gadget*    Overall digital health literacy    Having someone who may assist them using technologies | (s^2^: 32,0 / s: 5,6)  84%  92%      3,7 (±1,2)  3,2 (±1,4)  2,3 (±1,1)    1,1 (±0,9)    92% |

# Table 2 – Key features of applicable digital health solution

| **Identified unmet needs** | **Current services** | **Applicable digital solutions** |
| --- | --- | --- |
| **Attending preoperative consultations** | Healthcare professionals define a consultation calendar which is communicated to the patient orally and printed on a paper | Shared digital calendar, with alerts via SMS or mHealth app with personalized information (time, date, what to bring...) and indications on patient preparation (e.g. fasting hours) |
| **Polypharmacy management** | Health professionals draw up a personalized treatment plan on taking medications which is communicated orally to the patient and printed on a paper | Shared therapeutic plan, which can be updated remotely, with alerts on drug intake times and connected via Bluetooth to a smart pill box, which allows the patient to take the right drug, at the correct time and in the correct doses. |
| **Adherence to postoperative dressing prescriptions** | Healthcare professionals give the patient a sheet with post-operative recommendations and a checklist of actions to be carried out. Healthcare Professionals communicate orally dressing prescriptions. | Digital training package including a videolibrary with dressings tutorials and dressing prescriptions with guidelines and photos of the products they need and how to use them |
| **Follow up and recurrence prevention** | Health professionals provide a paper follow-up appointment reminder and tips for early detection of recurrences orally and through paper guidelines | Shared digital calendar, with alerts for follow-up appointments and a video tutorial for preventing recurrences, with key information and photo examples. A notification system promotes patient engagement in preventing relapses |
| **Easily accessible medical assistance** | Patients communicate their doubts and information requests by calling the hospital or writing directly to professionals via SMS. | A list of F.A.Q. is accessible by the patient with video tutorials and information materials. An AI-supported chatbot allows patients to quickly access the information they require. |

# Table 3 – Summary table of identified technological solutions

| **USE CASE DOMAINS** | **SUB-CATEGORY** | **IDENTIFIED UNMET NEEDS** | **CURRENT CLINICAL TASKS** | **FUNCTIONALITIES OF THE SUPPORTIVE DIGITAL SOLUTIONS** | **EXAMPLES** |
| --- | --- | --- | --- | --- | --- |
| Professionals/Carers Concerns | Communication and monitoring | Lack of continuous direct contact between patient and care team | Postoperative follow-up, treatment education | AI chatbot, educational videos, shared digital calendar | Integrated mHealth app with reminders and FAQs |
|  | Telemedicine | Need for remote consultations, especially in suburban areas | Post-op monitoring, easier access to professionals | Video calls, asynchronous messaging system | Secure telemedicine platform, virtual follow-up |
| Health concerns | Hospitalization and complications | Fear of prolonged hospital stay, infections, slow healing | Wound healing monitoring, complication management | Remote monitoring, digital educational packages | Photo documentation via app for wound tracking |
|  | Recurrence | Concern about future lesions or recurrence | Periodic dermatological follow-ups | Follow-up reminders, educational content on recurrence signs | App-based guided self-examination |
| Treatments | Therapy adherence | Forgetting medications or incorrect dosage | Medication management, follow-ups | Medication alerts, Bluetooth-connected pillbox, shared treatment plan | Bluetooth-connected pillbox with smart alerts |
|  | Postoperative care | Difficulty following dressing instructions and recommendations | Health education, self-care support | Video tutorials, personalized care instructions, FAQs | Illustrated material, app notifications |
| Daily Living | Daily organization | Difficulty remembering appointments, coordinating tasks | Reminders, daily planning | Shared calendar with alerts | App synchronized with caregiver's phone |
|  | Autonomy support | Loss of independence after surgery | Discharge planning and home recovery | Personalized reminders and recovery checklist | Recovery checklists integrated in app |
| What is important to Patient | Access to information | Need for clear, updated, and accessible medical information | Patient education and empowerment | Video library, interactive materials, FAQs | App with chatbot and visual self-guidance |
|  | Safety and continuity of care | Feeling abandoned after discharge | Maintaining contact with healthcare team | Asynchronous communication, remote monitoring | Messaging system with nurse/surgeon |
| Digital engagement and support | Digital support and navigation | Low confidence with tech use, reliance on caregiver support | App usage, tech onboarding | Simple UI, caregiver-assisted navigation | Basic digital health literacy; Tech support from family and caregivers |
| Own resources and assets | Geographic and social support | Support exists but not integrated | Caregiver involvement in care process | Shared app access, dual notifications | Suburban area; caregivers involved in tech use |

# Table 4 – Digital intervention data set

| **UNMET NEEDS** | **DATA** | **TOOLS** | **SETTINGS** | **INTEROPERABILITY** | **NOTES** |
| --- | --- | --- | --- | --- | --- |
| Improving accessibility to healthcare | Area of residence (urban, suburban, rural); device ownership (smartphone/tablet) | mHealth app, chatbot, video tutorials, telemedicine portal | Outpatient and home settings | Compatible with electronic health records | 92% own a smartphone/tablet; 84% broadband; 52% in suburban areas |
| Improving ability to self-manage disease | Therapy adherence; understanding medical instructions | Shared calendar, Bluetooth smart pillbox, digital education | Preoperative and postoperative settings | Sync with Bluetooth devices, data storage | Basic digital health literacy; Tech support from family and caregivers |
| Polypharmacy management and adherence | Medication intake routines, risk of mismanagement | Medication reminders, smart pillbox | Home setting | Pharmacy or EHR system integration | Frequent medication intake errors reported |
| Remote consultations (Telemedicine) | Need for medical follow-up without in-person visits | Video calls, secure messaging systems | Home and outpatient | Integration with EHRs and hospital platforms | Relevant due to geographic dispersion (suburban areas) |
| Adherence to a healthy lifestyle | Smoking, alcohol use, overweight | Educational videos, motivational content | Home | Optional connection to lifestyle tracking tools | Pino smokes, is overweight, drinks occasionally |
| Digital engagement and support | Low tech confidence, high reliance on others | User-friendly app, caregiver-linked access | Home | User-centered, adaptive design | Basic digital health literacy; Tech support from family and caregivers |
